# Mechanism of zedoary turmeric oil multi-components targeting S100A14 to reprogram astrocytes against tumor brain metastasis

**DOI:** 10.3389/fphar.2026.1851146

**Published:** 2026-06-15

**Authors:** Xue-Yu Wang, Jin-Ru Liu, Zeng-yuan Yao, Xia Yang, Zhuo-Hong Cai, Ming-Rui Wang, Wei-Xi Wang, Qin-Zhe Huang, Hong-Xiang Zhang, Zhong-Qiu Liu, Qian Feng, Cai-Jun Xie, Rong-Rong Zhang, Li-Jun Qiao

**Affiliations:** 1 International Institute for Translational Chinese Medicine, School of Pharmaceutical Sciences, Guangzhou University of Chinese Medicine, Guangzhou, China; 2 Department of Neurosurgery, The Second Affiliated Hospital of Guangzhou University of Chinese Medicine, Guangzhou, China

**Keywords:** brain metastasis, GFAP^+^ astrocytes, myeloid-derived suppressor cells, S100A14, zedoary turmeric oil

## Abstract

**Background:**

Zedoary Turmeric Oil (ZTO) is an active extract derived from the traditional Chinese medicine Ezhu, which is conventionally used to promote blood circulation, remove blood stasis, and relieve pain. Nevertheless, the therapeutic potential and underlying molecular mechanism of ZTO against tumor brain metastasis (BrM) remain poorly elucidated.

**Methods:**

The anti-BrM efficacy of ZTO was firstly assessed through S100A14-overexpress tuomor cells intracarotid injection BrM mice model. Concurrently, GC-MS analysis, computer simulation, along with *in vitro* experiments were employed to comprehensively investigate the mechanism by which ZTO targets S100A14 to suppress BrM.

**Results:**

Our study first identified ZTO as a promising candidate against S100A14-driven BrM. In mouse model, high-dose ZTO (200 mg/kg) significantly reduced *in vivo* brain BLI from 21465.73 ± 20757.36 to 1417.37 ± 2878.98 in 4T1-S100A14 breast cancer. ZTO also exhibited concentration-dependent BrM inhibition effects in LLC S100A14 lung cancer. ZTO remodeled the BrM microenvironment by regulating GFAP^+^ astrocytes via the NF-κB pathway, inhibiting migration, invasion, EMT, and MMPs. Additionally, ZTO dose-dependently suppressed the secretion of IL-6, CCL2, and CXCL1, thereby reducing recruitment of both P-MDSCs and M-MDSCs. Multi-analysis revealed that Curdione, Curzerene, Germacrone, Curcumenol, and Curcumenone directly bind S100A14, enhancing its thermal stability and resistance to protease hydrolysis. CETSA results confirmed thermal stabilization: Curzerene ΔTm = +22 °C, Germacrone ΔTm = +14.2 °C, Curdione ΔTm = +17.7 °C.

**Conclusion:**

ZTO exhibited potent anti-BrM efficacy and the mechanism involved multiple components synergistically targeting S100A14 to reprogram astrocytes, which in turn reduced the secretion of pro-inflammatory factors, thereby inhibiting the recruitment of MDSCs and reshaping the immunosuppressive microenvironment of the brain. Collectively, our study provides the first experimental evidence that the multiple bioactive components in ZTO act synergistically to target S100A14, offering a promising new direction for the development of anti-BrM therapeutic strategies.

## Graphical Abstract

**Figure:**
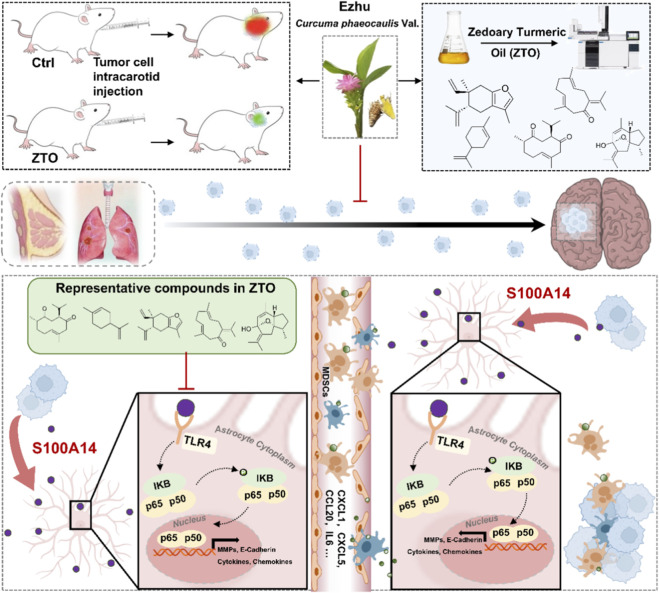

## 1 Introduction

Brain metastasis (BrM) represents one of the most devastating and formidable complications of advanced solid tumors, constituting a leading cause of cancer-related mortality (Achrol et al., 2019; Schulz and Prinz, 2025; Valiente et al., 2018). Epidemiological studies verified up to 50% of patients with lung cancer and 15%–35% of those with breast cancer will eventually develop to BrM during cancer disease progression (Hockemeyer et al., 2024; Sung et al., 2021; Xiao et al., 2020). The clinical management of BrM is profoundly challenging, fundamentally limited by the unique anatomical and physiological characteristics of the brain (Schulz and Prinz, 2025). A major therapeutic bottleneck lies in the poor blood-brain barrier (BBB) permeability of most conventional chemotherapeutic and targeted agents: for instance, drugs including doxorubicin, trastuzumab, and numerous tyrosine kinase inhibitors achieve only negligible concentrations in the brain parenchyma, leading to subtherapeutic exposure at metastatic foci and subsequent rapid treatment failure (Arvanitis et al., 2019; Schulz and Prinz, 2025). Despite significant advances in targeted and immunotherapeutic modalities, only a subset of patients with BrM exhibit encouraging remission responses in the initial stage, and these remissions are often short-lived and lack durability (Binnewies et al., 2018). Therefore, traditional therapies such as whole-brain radiotherapy (WBRT), stereotactic radiosurgery (SRS) and surgical resection, remain the cornerstone of the treatment for BrM. However, even with these interventions, the median overall survival for patients with BrM is only 4–8 months, which highlights the critical need for more effective and durable therapeutic strategies (Binnewies et al., 2018; Dagogo-Jack and Shaw, 2018; Lu et al., 2025).

The pathophysiological progression of BrM entails a multistep, complex cascade, ultimately determined by the dynamic interplay between circulating tumor cells and the unique microenvironment of the brain (Arvanitis et al., 2019; Qu et al., 2023). In recent years, increasing evidence has highlighted the critical involvement of astrocytes, the most abundant glial cells in the CNS and integral constituents of the blood-brain barrier (BBB), in facilitating the initiation and progression of BrM across various primary cancers (Arvanitis et al., 2019; Qu et al., 2023; Perelroizen et al., 2022; Quail and Joyce, 2017). Julien Sage et al. confirmed that astrocytes promote BrM of small cell lung cancer by secreting neuronal pro-survival factors such as SERPINE1 (Qu et al., 2023). Joan Massagué through single-cell transcriptomics of the tumor microenvironment revealed that triple-negative breast cancer cells tend to form perivascular sheaths with diffusive contact with astrocytes (Gan et al., 2024). Du Hua Yu et al. also verified that brain astrocytes cause the excessive expression of neuronal-specific cyclin-dependent kinase five in breast cancer-derived BrMs, thereby promoting the growth of BrM in mice (Yuzhalin et al., 2024). Despite these important observations, the precise mechanisms by which astrocytes are reprogrammed to create a “pre-metastatic niche” in the brain remain largely unexplored. In particular, how targeting astrocyte-tumor crosstalk might therapeutically reverse this supportive microenvironment constitutes a critical scientific gap in the BrM field.

The brain tissue is regarded as a special environment with “immune privilege”. When the permeability of the BBB is altered, peripheral immune cells can be specifically recruited into the brain to participate in the response (Louveau et al., 2015). An increasing evidence have shown that myeloid-derived suppressor cells (MDSCs), which are a heterogeneous population of immature myeloid cells with potent immunosuppressive activity, as the core coordinator of the immunosuppressive microenvironment in cancer (Li et al., 2021; Veglia et al., 2018). The latest research from Science utilized single-cell RNA sequencing and spatial transcriptomics to dissect the distribution of MDSC subsets within the tumor microenvironment of glioma and revealed a novel mechanism whereby MDSCs spatially co-localize with glioma stem-like cells, and their symbiotic interaction collaboratively drives tumor progression (Jackson et al., 2025). Our previously study also confirmed that both polymorphonuclear MDSCs (P-MDSCs) and monocytic MDSCs (M-MDSCs) play a significant role in immune suppression during tumor BrM.

S100A14, a 101-amino-acid protein with a molecular weight of approximately 11.5 kDa, was first identified in the year 2000 (Allgöwer et al., 2020; Bresnick 2018; Bresnick et al., 2015). Accumulating evidence has firmly established S100A14 upregulated in various human malignancies, including but not limited to esophageal, gastric, colorectal, lung, and breast cancers and as a significant player in tumorigenesis and cancer progression (Adam et al., 2025; Zhao et al., 2013; Zhu et al., 2021). For example, S100A14 is frequently upregulated in ovary and cervix malignancies and functions as an oncogenic driver, enhancing proliferation, invasion, metastasis, and epithelial-mesenchymal transition (EMT) through activation of key signaling pathways such as HER2/AKT/ERK, PI3K/AKT, and RAGE/NF-Κb (Chen et al., 2012; Hua et al., 2020; Li et al., 2020). Our previously research has confirmed that S100A14 represents a clinically relevant biomarker and therapeutic target for BrM. Specifically, S100A14 was highly expressed in the serum of lung cancer patients with BrM and exhibited a strong negative correlation with breast and lung cancer overall survival. We also verified S100A14 drives tumor BrM by targeting astrocytic TLR4, activating NF-κB signaling, reprogramming astrocytes to secrete pro-inflammatory cytokines (IL-6) and chemokines (CCL2 and CXCL1) to recruit MDSCs, and eventually shaping the brain pre-immunosuppressive niche. Thus, S100A14-mediated astrocyte reprogramming represents a key, targetable driver of the pre-metastatic microenvironment in BrM, and addressing this gap is the central focus of the present study. Therefore, developing therapeutic strategies targeting S100A14 for BrM treatment is highly promising.

Zedoary Turmeric Oil (ZTO) is an extract from Ezhu (the rhizome of Curcuma phaeocaulis Val.), a traditional herb historically used for promoting blood circulation and resolving stasis. Modern studies have demonstrated that ZTO and its components (e.g., curzerene, germacrone, curdione) possess broad-spectrum antitumor activities against lung cancer, colon cancer, melanoma, liver cancer, etc. (Barreto and Jandus, 2022; Giordano and Tommonaro, 2019; Lakshmi et al., 2011; Wang et al., 2023; Yang et al., 2025). However, whether ZTO exerts any effect against tumor brain metastasis remains completely unexplored.

In this study, we firstly elucidated the inhibitory role of Zedoary Turmeric Oil (ZTO), an extract from Ezhu, on tumor BrM utilizing an intracarotid injection BrM mouse model. In parallel, we also employed gas chromatography-mass spectrometry analysis, molecular docking, and molecular dynamics simulation, combined with cellular thermal shift assay and drug affinity responsive target stability experiments, to comprehensively demonstrate the underlying mechanism of ZTO in suppressing tumor BrM. Our study not only established a multi-level systematic methodological reference of “ingredient-target-pathway” for the research on the complex mechanism of TCM extracts, but also provided theoretical foundation for the potential clinical application of ZTO in the treatment of tumor BrM by targeting S100A14-mediated astrocyte reprogramming.

## 2 Materials and methods

### 2.1 Cell culture and compounds

Mouse breast cancer 4T1-luc cells were purchased from Beijing Bokai Biotechnology Co., Ltd. The mouse lung cancer Lewis-luc cells were purchased from Guangzhou Kain Biotechnology Co., Ltd. Both 4T1-luc cells and Lewis-luc cells were cultured in Dulbecco’s modified Eagle medium (DMEM) containing 10% fetal bovine serum (FBS) and 2 μg/mL puromycin at 37 °C and 5% CO_2_. The human lung cancer A549-luc cells were purchased from Shanghai Hengrui Biotechnology Co., Ltd. and cultured in RPMI-1640 containing 10% FBS and 2 μg/mL puromycin at 37 °C and 5% CO_2_. The human breast cancer MDA-MB-231-luc cells were purchased from Shanghai Obio Biotechnology Co., Ltd. and cultured in Leibovitz’s L15 containing 10% FBS and 2 μg/mL puromycin at 37 °C with 100% air. Purified recombinant S100A14 (#ATAP01971) were purchased from AtaGenix Biotechnology Technology (Wuhan, China) Co., Ltd. Temozolomide (TMZ) was purchased from Sigma-Aldrich. ZTO was purchased from Jiangxi Xinxen Natural Plant Oil Co., Ltd.

### 2.2 Primary immune cells isolation from tumor-bearing mice and flow cytometry

Mice were deeply anesthetized and transcardially perfused with ice-cold phosphate-buffered saline (PBS) until tissues cleared. Whole brains were harvested, minced, and enzymatically digested using a solution of Collagenase D (1 mg/mL) and DNase I (20–50 μg/mL) in Hank’s balanced salt solution (HBSS) at 37 °C for 15–30 min. The resulting single-cell suspension was filtered through a 70 μm strainer. Brain mononuclear cells were isolated via density gradient centrifugation using a pre-formed discontinuous Percoll (1#7-0891-01, GE Healthcare) gradient (30%/70%). After centrifugation at 800 ×g for 20 min (with no brake), the cells at the interface were collected and washed. For immunophenotyping, cells were stained with an antibody cocktail containing anti-Ly6G (1A8, APC-Cy7, #127624, BioLegend) and anti-Ly6C (HK1.4 APC, #17-5932-82, Invitrogen) for 30 min on ice, protected from light. Appropriate unstained and single-stained controls were included. Cells were washed, resuspended in PBS, and analyzed on a BD FACSAriaII flow cytometer.

### 2.3 Primary astrocytes isolation

Primary astrocytes were isolated from the cerebral cortex of postnatal (P0-P2) mice. Following rapid decapitation and disinfection, brains were aseptically dissected in ice-cold D-Hanks (#H1045, Solarbio) solution. Cortical tissues were meticulously freed of meninges and blood vessels, then digested with 0.25% trypsin-EDTA (#25200072, Thermo Fisher) at 37 °C for 15–20 min. Digestion was terminated by adding complete medium, and the tissue was triturated to obtain a single-cell suspension. After filtration through a 70 μm strainer, cells were pelleted by centrifugation (120 × g for 5 min). The cells were resuspended in complete medium and seeded onto poly-L-lysine-coated culture vessels at a density of 5 × 10^5^ cells/cm^2^. Cultures were maintained at 37 °C with 5% CO_2_. The medium was changed at 48 h post-seeding and subsequently every 2–3 days. To purify the astrocyte population, confluent cultures (days 7–10) were subjected to orbital shaking at 250 rpm for 18–24 h at 37 °C to remove loosely adherent microglia and oligodendrocyte precursors. Astrocyte purity was confirmed by immunofluorescence (IF) staining for the specific marker glial fibrillary acidic protein (GFAP), with DAPI counterstaining. Cultures routinely exhibited >95% GFAP-positive cells.

### 2.4 *In vitro* conditioned media co-culture experiments

Conditioned media were collected from S100A14-overexpressing tumor cells (A549-S100A14, LLC-S100A14, 231-S100A14, and 4T1-S100A14) following serum-free culture. Primary astrocytes were divided into experimental groups and treated for 24 h with: normal culture medium; CM from S100A14-overexpressing tumor cells; or CM supplemented with varying concentrations of ZTO (50, 100, and 200 μg/mL). Following treatment, supernatants from all astrocyte cultures were collected, centrifuged at 1,500 × g for 10 min at 4 °C to remove cellular debris, aliquoted, and stored at −80 °C. Astrocytes from all treatment groups were harvested for subsequent protein and RNA extraction.

### 2.5 MDSCs recruitment experiment

Bone marrow cells were harvested from the femurs and tibiae of euthanized mice. Following tissue removal and bone surface cleaning, marrow was flushed from the cavities using ice-cold complete medium. The cell suspension was filtered through a 70 μm strainer, centrifuged (300 × g, 5 min, 4 °C), and subjected to red blood cell lysis. After washing, cells were counted and resuspended to a concentration of 1 × 10^7^ cells/mL in PBS. For the transwell migration assay, cells were seeded into the upper chamber of transwell inserts at 3 × 10^5^ cells in 200 μL serum-free medium. The lower chambers contained 700 μL of one of the following: serum-free medium, CM from a tumor cell-astrocyte co-culture, or CM supplemented with ZTO (50, 100, or 200 ug/mL). After a 3 h incubation, migrated cells were collected from the lower chamber, washed, and stained with antibodies against Ly6G and Ly6C for 30 min on ice, protected from light. Following staining, cells were washed, resuspended in staining buffer, and analyzed by flow cytometry to quantify migrated myeloid cell subpopulations.

### 2.6 Intracarotid BrM mice model

The intracarotid injection model was used to evaluate the inhibitory effects of ZTO on BrM, according to our previous study (Feng et al., 2025; Feng et al., 2026). All mice were purchased from the Guangdong Medical Experimental Animal Center and all animal experiments were approved by the Animal Care and Use Committee of Guangzhou University of Chinese Medicine (SYXK-2024-0144 Animal Use License). 4-6 weeks-old BALB/c or C57BL/6 mice were anesthetized. Under aseptic conditions, a midline neck incision was made to expose the carotid artery. The proximal common carotid artery was ligated, and 5 × 10^3^ 4T1-S100A14-luc cells or 2 × 10^5^ LLC cells (in 100 µL suspension) were slowly injected into the artery using a 31-33G needle. The distal artery was occluded after injection to prevent reflux, and the wound was closed. Three days post-inoculation, mice were randomly allocated into five groups (n = 8 per group): Model (0.05% Tween 80 vehicle), TMZ (25 mg/kg), and Low-, Medium-, and High-dose ZTO (50, 100, and 200 mg/kg, respectively). ZTO was dissolved in 0.05% Tween 80 solution. Treatments were administered for 2 weeks. Body weight and neurological status were monitored daily. Tumor growth was assessed periodically via *in vivo* bioluminescence intensity (BLI) or other appropriate methods. Mice were euthanized when body weight fell below 15 g or at the experimental endpoint. *Ex vivo* analysis of the brain was performed post-dissection.

### 2.7 Wound healing assay

Primary astrocytes were harvested by trypsinization, resuspended, and counted. The cell suspension was seeded into 6-well plates at a density of 3 × 10^6^ cells per well. After complete cell adhesion, a uniform straight scratch was created using a sterile 10 μL pipette tip perpendicular to the plate bottom along pre-marked lines, maintaining consistent pressure throughout the procedure. The old medium was removed, and the cells were gently washed 2-3 times with pre-warmed PBS to remove dislodged cellular debris. The medium was then replaced with one of the following test conditions: serum-free medium; serum-free CM from S100A14-overexpressing tumor cell-astrocyte co-culture; The same CM supplemented with ZTO at concentrations of 50, 100, or 200 μg/mL. The plate was placed on an inverted microscope stage, and wound areas with clear boundaries were identified under 10× objective magnification. These locations were marked on the bottom of the plate to ensure consistent imaging positions. Multiple random fields within each wound area were photographed and recorded as the 0-h time point. The plate was returned to a 37 °C, 5% CO_2_ incubator for continued culture. At predetermined time points post-scratch (24 h and 48 h, based on cell migration rate), the plate was removed and images were captured at the previously marked identical locations. Images from different time points were analyzed using ImageJ software.

### 2.8 Transwell migration assay

Primary astrocytes were seeded into 6-well plates at a density of 3 × 10^5^ cells per well. Upon reaching confluence, a uniform linear scratch wound was created using a sterile 200 μL pipette tip. After washing with PBS to remove detached cells, the medium was replaced with one of the following serum-free test media: control medium, CM from S100A14-overexpressing tumor cell-astrocyte co-culture, or the same CM supplemented with ZTO at final concentrations of 50, 100, or 200 μg/mL. Immediately after scratching (0 h), multiple pre-marked fields per well were imaged under a 10×phase-contrast objective. The plates were then returned to the incubator (37 °C, 5% CO_2_) for continued culture. Wound closure was monitored, and images of the same fields were captured again at 24 h and 48 h post-scratch. The migration rate was quantified by measuring the change in wound area using ImageJ software.

### 2.9 Molecular docking analysis

The crystal structure of human S100A14 protein (PDB ID: 2M0V) was retrieved from the Protein Data Bank and prepared in PyMOL by removing water molecules and co-crystallized ligands. The complete chemical profile of ZTO was determined by Gas Chromatography-Mass Spectrometry (GC-MS). All identified constituents were subsequently used as ligands for semi-flexible molecular docking against the S100A14 protein using AutoDock Vina 1.1.2. The protein structure was kept rigid, while all rotatable bonds in each ligand were allowed free rotation. For each compound, the conformation with the most favorable binding free energy (ΔG) was selected. Molecular interactions, including hydrogen bonds, hydrophobic contacts, π-alkyl/π-cation interactions, and salt bridges between ligands and specific residues in the S100A14 binding pocket, were characterized and visualized using PyMOL.

### 2.10 Molecular dynamics (MD) simulation

The S100A14-ligand complex with the most favorable binding free energy from the docking analysis was selected as the starting structure for MD simulation. The apo-protein was simulated in parallel as a control. The simulation system was prepared using the GROMACS software package. The complex was solvated in a TIP3P water box with a 10 Å buffer and neutralized by adding Na^+^ and Cl^−^ ions to a final concentration of 150 mM NaCl. The CHARMM36 force field was applied to the protein, and the CGenFF parameters were assigned to the ligand. The system first underwent energy minimization via the steepest descent algorithm, followed by stepwise equilibration under the NVT and NPT ensembles. Subsequently, a 100 ns production MD simulation was performed under constant temperature (310K) and pressure (1bar) conditions, with trajectory coordinates saved every 100 ps. The resulting trajectories were analyzed for stability and dynamics. The root-mean-square deviation (RMSD) of the protein backbone and the root-mean-square fluctuation (RMSF) of individual residues were calculated to assess global conformational stability and local flexibility, respectively.

### 2.11 Cellular thermal shift assay (CETSA)

A549-S100A14 cells were treated with test compounds (50 µM) or vehicle (0.1% DMSO) for 3 h. Following treatment, cells were harvested, washed with PBS, and resuspended in PBS containing protease inhibitors at a density of 1 × 10^7^ cells/mL. Aliquots of the cell suspension were subjected to a temperature gradient (40 °C–70 °C, 5 °C intervals) for 5 min each using a thermal cycler, followed by immediate cooling on ice. The heat-treated samples were lysed with RIPA buffer, and the clarified supernatants were collected after centrifugation. The total protein concentration of each supernatant was determined using the BCA assay and adjusted to 1 mg/mL with RIPA buffer. Protein concentrations were normalized, and equal amounts (20 µg) of protein from each temperature point were separated by SDS-PAGE and transferred to PVDF membranes. Membranes were blocked and incubated overnight at 4 °C with an anti-S100A14 primary antibody (1:1000), followed by incubation with an HRP-conjugated secondary antibody (1:10000). Target protein bands were visualized using enhanced chemiluminescence (ECL), and their relative intensities at different temperatures were quantified by densitometry using ImageJ software to generate thermal stability curves.

### 2.12 Drug affinity responsive target stability assay (DARTS)

A549-S100A14 cells were treated with 50 µM test compounds (from ZTO) or vehicle (0.1% DMSO) for 3 h. Cells were then lysed, the total protein concentration of the lysates was determined using the BCA method and adjusted to 2 mg/mL, and the resulting cell lysates were divided into equal aliquots. Each aliquot was subjected to proteolysis by incubation with varying concentrations of pronase (at ratios ranging from 1:125 to 1:4000, with one sample as a no-enzyme control) for 10 min at room temperature. The proteolytic reaction was stopped by adding 1 mM PMSF. The protein samples were then separated by SDS-PAGE and transferred to PVDF membranes. The membranes were immunoblotted using an anti-S100A14 primary antibody (1:1000) and an HRP-conjugated secondary antibody (1:5000). Target protein bands were visualized using enhanced chemiluminescence (ECL), and their intensities were quantified with ImageJ software to assess the compound-induced stabilization of S100A14 against proteolysis.

### 2.13 Enzyme-linked immunosorbent assay (ELISA)

Concentrations of IL-6, CCL2, and CXCL1 in cell culture supernatants and mouse serum were determined using commercial ELISA kits. After sample and standard incubation, plates were sequentially incubated with biotinylated detection antibodies and HRP-conjugated streptavidin. Reactions were developed with TMB substrate, stopped, and absorbance was measured at 450 nm. Cytokine concentrations were calculated from standard curves.

### 2.14 Western blot analysis

Protein was extracted from astrocytes or brain tissues using RIPA buffer containing protease and phosphatase inhibitors. Protein concentrations were determined using the BCA method (Thermo Scientific, #23225) and equal amounts (20–40 μg) of protein per sample were separated by SDS-PAGE and transferred to PVDF membranes. Membranes were blocked with 5% non-fat milk in TBST for 1 h at room temperature, then probed overnight at 4 °C with primary antibodies against NF-κB pathway components: anti-NF-κB (clone D14E12, 1:1000, Cell Signaling Technology, #8242), anti-p-NF-κB (Ser536, clone 93H1, 1:1000, CST #3033), anti-IKKβ (clone D30C6, 1:1000, CST #8943), anti-p-IKKα/β (Ser176/180, clone 16A6, 1:1000, CST #2697), anti-p-IκBα (Ser32, clone 14D4, 1:1000, CST #2859), and anti-GAPDH (clone 6C5, 1:5000, Abcam #ab8245). After incubation with HRP-conjugated secondary antibodies (goat anti-rabbit IgG, 1:10000, CST #7074; or goat anti-mouse IgG, 1:10000, CST #7076) for 1 h at room temperature, protein bands were visualized using enhanced chemiluminescence (ECL, Millipore, #WBKLS0500) and quantified with ImageJ software. The relative protein expression levels were normalized to GAPDH.

### 2.15 Quantitative real-time PCR (qPCR)

Total RNA was extracted from astrocytes or brain tissues using TRIzol reagent. cDNA was synthesized from 1 µg of RNA. qPCR was performed using SYBR Green chemistry and gene-specific primers for IL-6, CCL2, CXCL1, and other targets, with GAPDH as the reference gene. Relative mRNA expression levels were calculated using the 2^(–ΔΔCt)^ method. All primer sequences were shown in Table 1.

**TABLE 1:** Primers list.

Gene	Forward primer (5′-3′)	Reverse primer (5′-3′)
CXCL10	CCA​AGT​GCT​GCC​GTC​ATT​TTC	GGC​TCG​CAG​GGA​TGA​TTT​CAA
CX3CL1	ACG​AAA​TGC​GAA​ATC​ATG​TGC	CTG​TGT​CGT​CTC​CAG​GAC​AA
IL-6	GAT​GCA​ACC​AAA​CTG​GAT​ATA​ATC	GAG​CAT​TGG​AAG​TTG​GGG​TA
CCL22	AGG​TCC​CTA​TGG​TGC​CAA​TGT	CGG​CAG​GAT​TTT​GAG​GTC​CA
CXCL16	CCT​TGT​CTC​TTG​CGT​TCT​TCC	TCC​AAA​GTA​CCC​TGC​GGT​ATC
CCL11	GAA​TCA​CCA​ACA​ACA​GAT​GCA​C	ATC​CTG​GAC​CCA​CTT​CTT​CTT
CXCL11	GGC​TTC​CTT​ATG​TTC​AAA​CAG​GG	GCC​GTT​ACT​CGG​GTA​AAT​TAC​A
CCL5	ATA​TGG​CTC​GGA​CAC​CAC​TC	TTC​GAG​TGA​CAA​ACA​CGA​CTG
CXCL5	GCC​CCT​TCC​TCA​GTC​ATA​GC	AGC​TTT​CTT​TTT​GTC​ACT​GCC​C
CCL2	CAC​TCA​CCT​GCT​GCT​ACT​CA	TGA​GCT​TGG​TGA​CAA​AAA​CTA​CAG
β-actin	CACTGTCGAGTCGCGTCC	TCA​TCC​ATG​GCG​AAC​TGG​TG

### 2.16 Hematoxylin and eosin and IF stain

Brain tissues were fixed in 4% paraformaldehyde, paraffin-embedded, and sectioned at 4–5 μm thickness. For H&E staining, deparaffinized and rehydrated sections were sequentially stained with hematoxylin and eosin, followed by dehydration, clearing, and mounting. Histopathological analysis was performed under a light microscope.

For IF staining, after antigen retrieval in citrate buffer and permeabilization with 0.3% Triton X-100, sections were blocked with 3% BSA. Sections were then incubated overnight at 4 °C with a primary antibody against the astrocyte marker GFAP. Following washes, sections were incubated with the appropriate fluorophore-conjugated secondary antibody and counterstained with DAPI. Images were captured using a laser scanning confocal microscope. The mean fluorescence intensity of GFAP in the tumor microenvironment was quantified using ImageJ software to evaluate astrocyte activation.

### 2.17 GC-MS/MS analysis of ZTO

The chemical composition of ZTO was analyzed using an Agilent 7890A gas chromatograph coupled with a 5977A mass spectrometer (Agilent Technologies, Santa Clara, CA, United States). Separation was performed on a HP-5 capillary column (30 mm × 0.25 mm × 0.25 µm). High-purity helium was used as the carrier gas at a constant flow rate of 15 mL/min. The sample (5 μL) was injected in split mode (split ratio 10:1) with the injector temperature set at 250 °C. The oven temperature was programmed as follows: initial hold at 60 °C for 3 min, increased to 80 °C at a rate of 1.5 °C/min, then raised to 120 °C at 4 °C/min, followed by an increase to 180 °C at 5 °C/min, and finally ramped to 260 °C at 10 °C/min, with a final hold for 3 min. Electron ionization (EI) mass spectra were acquired in full-scan mode (m/z 30–500) with an ionization energy of 70 eV. The ion source and transfer line temperatures were maintained at 230 °C and 280 °C, respectively, with a solvent delay of 3 min. Identification of major components was performed by comparing the mass spectra with the NIST mass spectral library (version 2.0). A component was considered identified when the matching score was ≥85%. The main components identified in ZTO included curzerene, germacrone, curdione, β-elemene, curcumol, and neocurdione.

### 2.18 Statistical analysis

All experimental data are presented as mean ± standard deviation (SD) or median with interquartile range (IQR) as indicated. Normality of data distribution was assessed using the Shapiro-Wilk test. Homogeneity of variances was evaluated using Levene’s test. Statistical analyses were performed using GraphPad Prism software (version 9.0). For data that followed a normal distribution and exhibited homogeneity of variance, parametric tests were applied: for comparisons between two groups, an unpaired Student's t-test was used; for comparisons among multiple groups, one-way analysis of variance (ANOVA) was employed, followed by Tukey’s *post hoc* test for pairwise comparisons. For data that did not pass the normality test or showed unequal variances, non-parametric tests were used: the Mann-Whitney U test for two-group comparisons and the Kruskal-Wallis H test followed by Dunn’s *post hoc* test for multiple-group comparisons. All statistical tests were two-sided, and a probability value of P < 0.05 was considered statistically significant. Significance levels in figures are denoted by asterisks: (P < 0.05), (P < 0.01), (P < 0.001), and (****P < 0.0001).

## 3 Results

### 3.1 ZTO significantly inhibited S100A14-induced BrM in mouse intracarotid injection model

**FIGURE 1:**
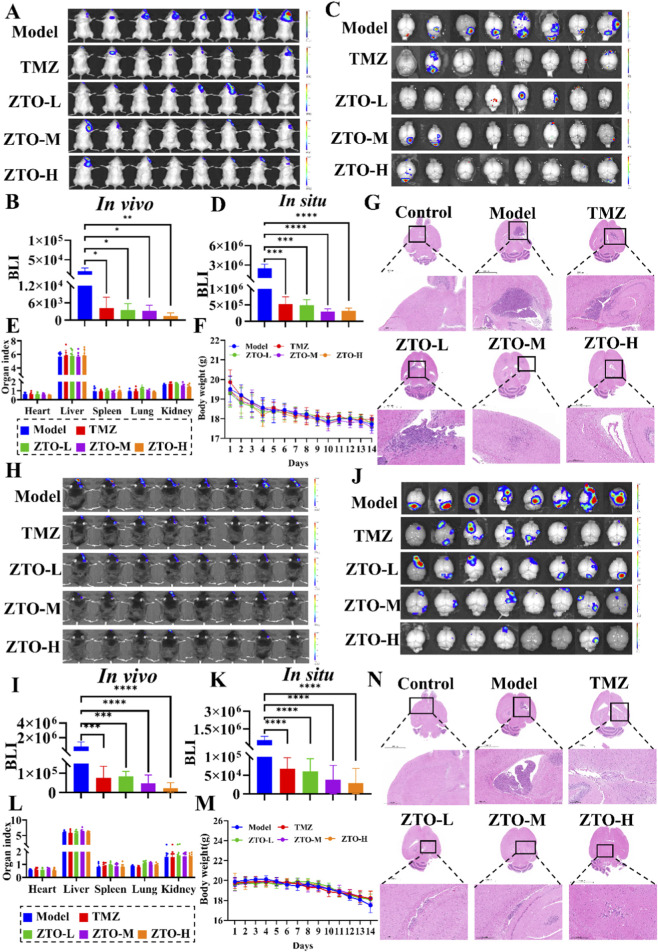
ZTO significantly inhibited S100A14-induced BrM in mouse intracarotid injection model. *In vivo* **(A)** and corresponding quantification **(B)** of BLI in mice bearing 4T1-S100A14 with or without ZTO treatment. *In situ* **(C)** and corresponding quantification **(D)** of BLI in mice bearing 4T1-S100A14 with or without ZTO treatment. Organ index **(E)** and body weight **(F)** of mice bearing 4T1-S100A14 in each group. **(G)** HE staining of brain tissue in 4T1-S100A14 bearing mice in each group. *In vivo* **(H)** and corresponding quantification **(I)** of BLI in mice bearing 4T1-S100A14 with or without ZTO treatment. *In situ* **(J)** and corresponding quantification **(K)** of BLI in mice bearing 4T1-S100A14 with or without ZTO treatment. Organ index **(L)** and body weight **(M)** of mice bearing 4T1-S100A14 in each group. **(N)** HE staining of brain tissue in 4T1-S100A14 bearing mice in each group. Data represent mean ± SD.(n = 8) Significant difference versus control group, *P < 0.05, **P < 0.01, and ***P < 0.001.

To evaluate the therapeutic effect of ZTO on tumor BrM, we established S100A14-overexpressing breast cancer (4T1) and lung cancer (LLC) BrM models via intracarotid artery injection. As shown in Figures 1A,C, compared to the model group, ZTO treatment significantly reduced 4T1-S100A14 breast cancer BrM in a dose-dependent manner (P < 0.05 vs. model). *In vivo* BLI revealed that the photon fluxes in all ZTO treatment groups (50, 100, and 200 mg/kg) were significantly lower than that of the model group (P < 0.05 for 50 mg/kg; P < 0.01 for 100 and 200 mg/kg). Consistently, *ex vivo* fluorescence imaging of brain tissues confirmed the concentration-dependent inhibitory effect of ZTO on 4T1-S100A14 BrM (P < 0.05 vs. model; Figures 1B,D). These data demonstrate that ZTO possesses potent anti-BrM activity against 4T1-S100A14 breast cancer BrM *in vivo*, with the high-dose ZTO group exhibiting superior efficacy compared to TMZ. In addition, there was no significant body weight (Figure 1F) and organ index (Figure 1E) changes in all groups of mice, indicating that ZTO intervention had no obvious toxicity or adverse reactions to the body. To further validate the anti-BrM effect of ZTO, we performed H&E staining on brain tissues from the different treatment groups. As illustrated in Figure 1G, massive proliferation of 4T1-S100A14 cells were observed in the brain of the model group, characterized by well-demarcated tumor boundaries and marked nuclear atypia, including enlarged nuclei and increased nuclear-to-cytoplasmic ratios. In contrast, 100 mg/kg, and 200 mg/kg ZTO treatment groups exhibited a dramatic reduction in tumor burden, with scarcely any proliferating tumor cells infiltration in 200 mg/kg ZTO treatment group. Collectively, these results demonstrate that ZTO significantly inhibits 4T1-S100A14 breast cancer BrM.

Similar results were observed in the LLC-S100A14 lung cancer BrM model. ZTO treatment led to a significant decrease in tumor burden both *in vivo* (P < 0.05 vs. model; Figures 1H,J) and *ex vivo* (P < 0.05 vs. model; Figures 1I,K). Body weight (Figure 1M) and organ index (Figure 1L) data confirmed no obvious toxicity after 2 weeks of ZTO administration. H&E staining results consistently demonstrated that ZTO effectively inhibited LLC-S100A14 lung cancer BrM. Above all, our findings indicate that ZTO robustly inhibits BrM f both S100A14-overexpressing breast and lung cancers without eliciting significant adverse effects, highlighting its potential as a promising therapeutic agent for treating tumor BrM.

### 3.2 ZTO regulated NF-κB signaling of GFAP^+^ astrocytes to reduce pro-inflammatory cytokine production and remodel brain immunosuppressive microenvironment

**FIGURE 2:**
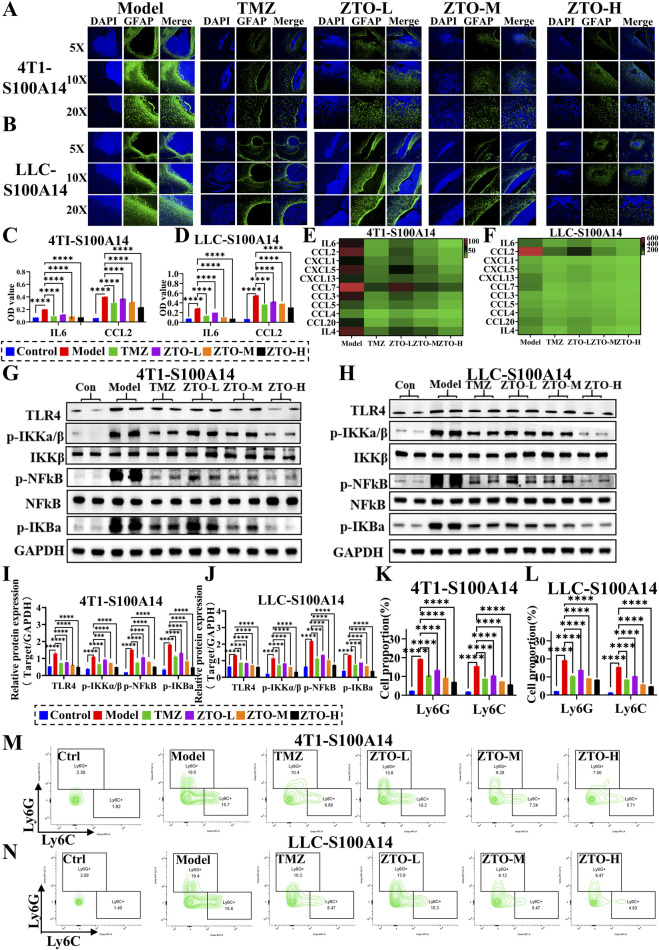
ZTO modulated NF-κB signaling in GFAP^+^ astrocytes to attenuate pro-inflammatory cytokine production and reprogram brain immunosuppressive microenvironment. IF co-staining of GFAP (green) and nuclei (DAPI, blue) were performed in brain tissues of mice bearing 4T1-S100A14 **(A)** or LLC-S100A14 **(B)** tumors with or without ZTO treatment. Scale bar: 50 µm. ELISA was used to detect the concentrations of IL6 and CCL2 in the plasma of 4T1-S100A14 **(C)** and LLC-S100A14 **(D)** bearing mice in each group. qRT-PCR analysis was used to detect the mRNA expression levels of cytokines and chemokines in astrocytes of 4T1-S100A14 **(E)** and LLC-S100A14 **(F)** bearing mice in each group. Western blot analysis and quantification of key protein expression levels of NF-κB signaling pathway in astrocytes of 4T1-S100A14 **(G,I)** and LLC-S100A14 **(H,J)** bearing mice in each group. Flow cytometric analysis and quantification of immune cells in brain tissue from 4T1-S100A14 **(M,K)** and LLC-S100A14 **(N,L)** bearing mice in each group. Data represent mean ± SD.(n = 3) Significant difference versus control group, *P < 0.05, **P < 0.01, and ***P < 0.001.

To elucidate the underlying mechanism of ZTO in inhibiting tumor BrM, we performed IF staining on brain tissues to assess. Our results revealed substantial enrichment of GFAP^+^ astrocytes surrounding tumor lesions in BrM model group. In contrast, both TMZ and ZTO treatments significantly reduced the accumulation of GFAP^+^ astrocytes. This reduction effects were consistently observed in both the 4T1-S100A14 breast cancer (Figure 2A) and LLC-S100A14 lung cancer (Figure 2B) BrM models. Concurrently, ELISA analysis of plasma samples revealed a systemic inflammatory response in tumor-bearing mice. Compared to normal controls, plasma levels of the pro-inflammatory cytokine IL-6 and the chemokine CCL-2 were markedly elevated in both the 4T1-S100A14 (Figure 2C) and LLC-S100A14 (Figure 2D) models. Notably, ZTO treatment dose-dependently reduced the plasma concentrations of both IL-6 and CCL2. Importantly, the 200 mg/kg ZTO treatment group exhibited markedly lower plasma levels of IL-6 and CCL2 in both tumor models compared to the positive control TMZ group (P < 0.05).

Subsequently, we isolated astrocytes from tumor-bearing mice using magnetic bead-based separation and performed qRT-PCR and western blot. qRT-PCR results demonstrated that ZTO treatment significantly downregulated the mRNA expression of key cytokines and chemokines in isolated astrocytes. In the 4T1-S100A14 model (Figure 2E), ZTO markedly reduced the expression of CCL7, CCL2, IL-4, and IL-6 compared to the model group (P < 0.05). Similarly, in LLC-S100A14 model (Figure 2F), ZTO significantly reduced the mRNA expressions of CCL7, CCL2, IL-4, and IL-6. Given that NF-κB signaling pathway plays a crucial role in the inflammatory response mediated by astrocytes, we next used western blotting to detect the activation of NF-κB signaling pathway. Western blot analysis revealed a marked activation of the NF-κB signaling pathway in astrocytes from the model groups, evidenced by markedly increased phosphorylation of IKKα/β, IκBα, and NF-κB (Figures 2G–J). Conversely, ZTO treatment dose-dependently decreased the levels of p-IKKα/β, p-IκBα, and p-NF-κB.

Finally, we analyzed immune cells isolated from the brain tissues by flow cytometry. The results confirmed a significant infiltration of both P-MDSCs and M-MDSCs in the brains of model mice compared to normal control (P < 0.05; Figures 2M,N). ZTO treatment significantly reduced the proportions of both P-MDSCs and M-MDSCs in the brain tissue in a dose-dependent manner. (Figures 2K,L). Collectively, these findings demonstrate that ZTO suppressed BrM by modulating the NF-κB signaling pathway in GFAP^+^ astrocytes, thereby suppressing the secretion of pro-inflammatory cytokines and chemokines, and subsequently inhibiting the recruitment of immunosuppressive MDSCs.

### 3.3 ZTO significantly inhibited the invasion and migration of astrocytes

**FIGURE 3:**
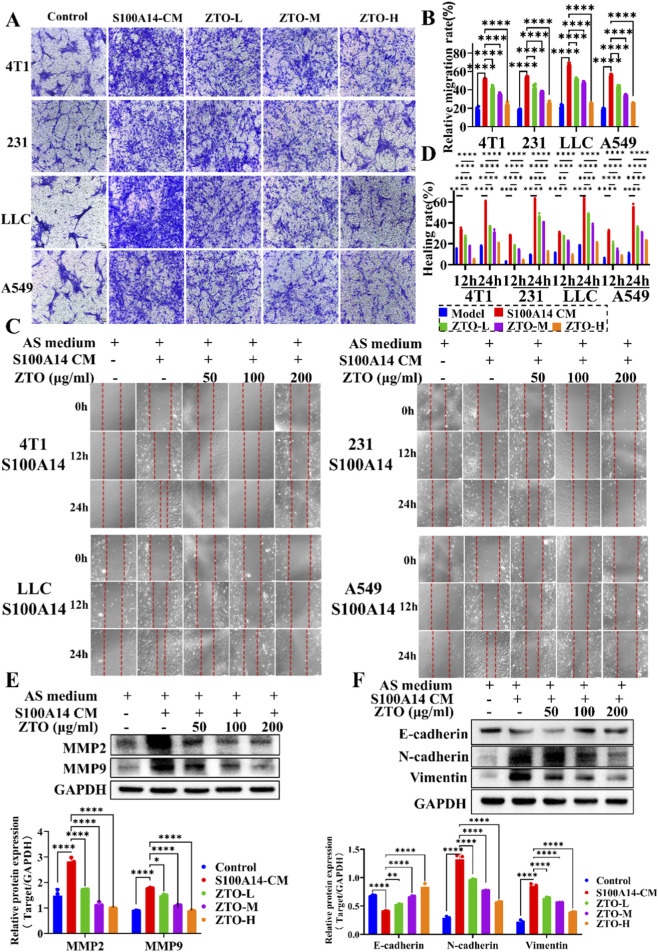
ZTO significantly suppressed the invasion and migration of astrocytes. The images **(A)** and quantification **(B)** of astrocytes induced by CM of S100A14-overexpressing tumor cells (4T1, MDA-MB-231, LLC, and A549) in the presence or absence of ZTO using the transwell migration assay. The images **(C)** and quantification **(D)** of astrocytes induced by CM of S100A14-overexpressing tumor cells (4T1, MDA-MB-231, LLC, and A549) in the presence or absence of ZTO using the wound healing assay. **(E)** Western blot analysis and quantification of MMP2 and MMP9 expression levels in astrocytes induced by CM of S100A14-overexpressing tumor cells (4T1, MDA-MB-231, LLC, and A549) in the presence or absence of ZTO. **(F)** Western blot analysis and quantification of key EMT-related proteins expression levels in astrocytes induced by CM of S100A14-overexpressing tumor cells (4T1, MDA-MB-231, LLC, and A549) in the presence or absence of ZTO. Data represent mean ± SD of three independent experiments.(n = 3) Significant difference versus control group, *P < 0.05, **P < 0.01, and ***P < 0.001.

Our preceding findings demonstrated substantial enrichment of GFAP^+^ astrocytes around brain metastatic lesions, a phenomenon markedly attenuated by ZTO treatment. We therefore hypothesized that ZTO has the ability to directly inhibit invasion and migration of astrocytes. Therefore, transwell migration and wound healing assays were performed. As shown in Figures 3A,B, CM harvested from S100A14-overexpressing cancer cells (4T1, MDA-MB-231, LLC, and A549) significantly enhanced the migration rate of astrocytes compared to the control (P < 0.05). Wound healing assays corroborated these findings, demonstrating that ZTO treatment accelerated wound closure in a concentration-dependent manner, further indicating the inhibitory effects of ZTO in astrocytes (Figures 3C,D). Furthermore, Western blot analysis revealed that S100A14-CM upregulated the protein levels of both MMP-2 and MMP-9. Conversely, ZTO treatment robustly suppressed the expression of MMP-2 and MMP-9 in a dose-dependent manner on astrocytes (Figure 3E). Then, the epithelial-mesenchymal transition (EMT) related proteins in Astrocytes were also detected by Western blotting. The results revealed that exposure to S100A14-CM downregulated the epithelial marker E-cadherin and upregulated the mesenchymal markers N-cadherin and Vimentin, indicating the induction of EMT in astrocytes. Notably, ZTO treatment effectively reversed these protein expression changes, thereby suppressing the S100A14-CM-driven EMT process (Figure 3F). These *in vitro* results consistent with our previously *in vivo* observations, in which S100A14-overexpressing tumor cells promoted a pronounced accumulation of astrocytes at the metastatic site, this effect that was markedly attenuated by ZTO administration. Collectively, these data demonstrate that ZTO inhibited the EMT-like transformation of astrocytes, likely through downregulation of MMP-2 and MMP-9 expression, resulting in suppressed migration and invasion of astrocytes.

### 3.4 ZTO reprogrammed astrocytes to suppress pro-inflammatory cytokine secretion and MDSCs recruitment

**FIGURE 4:**
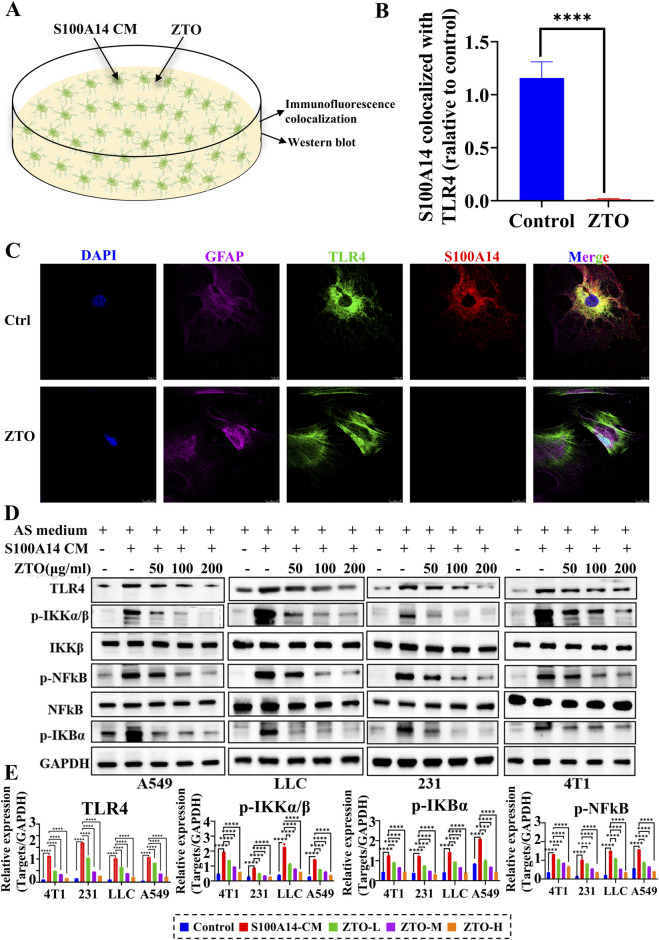
ZTO significantly inhibited the binding of S100A14 to the TLR4 in astrocytes to suppress the NF-κB signaling pathway. Schematic diagram of co-culture of ZTO with primary astrocytes and S100A14-CM **(A)**. The fluorescence co-localization **(C)** and columnar statistical results **(B)** of S100A14 and TLR4 in the primary astrocytes treated with ZTO. Western blot analysis **(D)** and quantification **(E)** of key protein expression levels of NF-κB signaling pathway in primary astrocytes treated with CM from S100A14 overexpression breast tumor cells (4T1, MDA-MB-231) and lung cancer cells (LLC, A549), with or without ZTO. Data represent mean ± SD of three independent experiments. Significant difference versus control group, *P < 0.05, **P < 0.01, and ***P < 0.001.

In order to further explore the mechanisms by which ZTO inhibited tumor-BrM in astrocytes, we isolated primary astrocytes and incubated them with different concentrations of ZTO (Figure 4A). The results showed that ZTO could significantly inhibit the binding of S100A14 to TLR4 (Figures 4C,D). Western blot analysis revealed a marked activation of the NF-κB signaling pathway in astrocytes in the S100A14-CM treatment groups, evidenced by markedly increased phosphorylation of IKKα/β, IκBα, and NF-κB (Figure 4D). Conversely, ZTO treatment effectively reversed NF-κB signaling pathway activation, leading to a dose-dependent decrease in phosphorylation protein levels of key pathway components, including IKKα/β, IκBα, and NF-κB p65 (Figure 4D). Collectively, our findings demonstrate that ZTO suppressed BrM by modulating the NF-κB signaling pathway in GFAP^+^ astrocytes.

**FIGURE 5:**
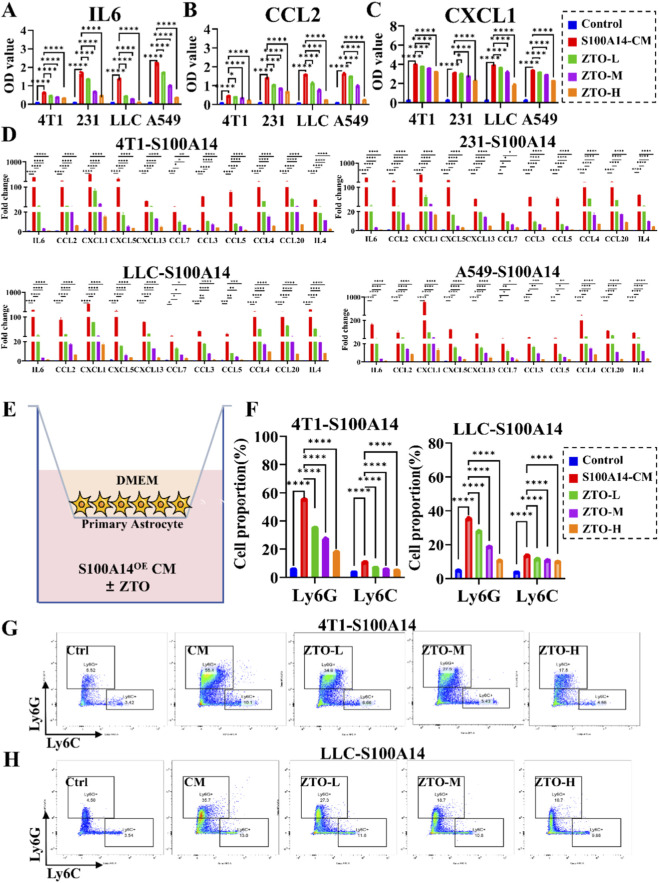
ZTO reprogrammed astrocytes to suppress pro-inflammatory cytokine secretion and MDSCs recruitment. Concentrations of IL6 **(A)**, CCL2 **(B)** and CXCL1 **(C)** in primary astrocytes treated with CM from S100A14 overexpression breast tumor cells (4T1, MDA-MB-231) and lung cancer cells (LLC, A549), with or without ZTO. **(D)** qRT-PCR analysis to detect the mRNA expression levels of cytokines (IL6, IL4) and chemokines (CCL2, CXCL1, CXCL5, CXCL13, CCL7, CCL3, CCL5, CCL4 and CCL20) in primary astrocytes treated with CM from S100A14 overexpression breast tumor cells (4T1, MDA-MB-231) and lung cancer cells (LLC, A549), with or without ZTO. **(E)** Schematic diagram of *ex vivo* MDSCs chemoattraction assay. **(F)** Statistical results of *ex vivo* MDSCs chemoattraction assay. Flow cytometric analysis and quantification of MDSCs recruitment in response to 4T1-S100A14 CM with or without ZTO **(G)** or LLC-S100A14 CM with or without ZTO **(H)**. Data represent mean ± SD (n = 3). Significant difference versus control group, *P < 0.05, **P < 0.01, and ***P < 0.001.

Our *in vivo* data demonstrated that ZTO administration significantly reduced the accumulation of both GFAP^+^ astrocytes and MDSCs in brains of tumor-bearing mice. To elucidate the underlying mechanism, we firstly established CM-primary astrocytes co-culture system (Figure 4A). ELISA results showed that CM harvested from S100A14-overexpressing breast (4T1, MDA-MB-231) and lung (LLC, A549) cancer cells significantly induced the secretion of the pro-inflammatory cytokine IL-6 and the chemokines CCL2 and CXCL1 from primary astrocytes (P < 0.05 vs. control), consistent with our *in vivo* observations. Strikingly, ZTO treatment dose-dependently suppressed S100A14-CM-induced secretion of IL-6, CCL2, and CXCL1 (P < 0.05 vs. CM alone; Figures 5A–C). Furthermore, qRT-PCR analysis revealed that ZTO treatment markedly downregulated the mRNA expression of a broad spectrum of cytokines (IL-6, IL-4) and chemokines (CCL2, CCL3, CCL4, CCL5, CCL7, CCL20, CXCL1, CXCL5, CXCL13) in primary astrocytes compared to the S100A14-CM group (Figure 4D). These data collectively confirm that ZTO reprogrammed astrocytes by modulating the NF-κB pathway, thereby suppressing the production of pro-inflammatory mediators.

To directly investigate the functional consequence of this reprogramming on the tumor microenvironment, we performed an *ex vivo* MDSCs chemoattraction assay (Figure 5E). Our results showed that S100A14-CM from both 4T1 and LLC cells robustly recruited both P-MDSCs and M-MDSCs (Figures 5G,H). Crucially, ZTO treatment dose-dependently and significantly inhibited the S100A14-CM-mediated recruitment of both P-MDSC and M-MDSC subsets (P < 0.05 vs. CM alone), demonstrating that ZTO disrupted the crosstalk between astrocytes and MDSCs. In summary, our findings verify that mechanism of ZTO in inhibiting tumor BrM was through regulating the NF-κB signaling pathway to reprogram astrocytes, inhibiting the release of pro-inflammatory cytokines and chemokines, ultimately preventing the recruitment of immunosuppressive MDSCs.

### 3.5 Identification of bioactive components in ZTO that directly targeting S100A14

**FIGURE 6:**
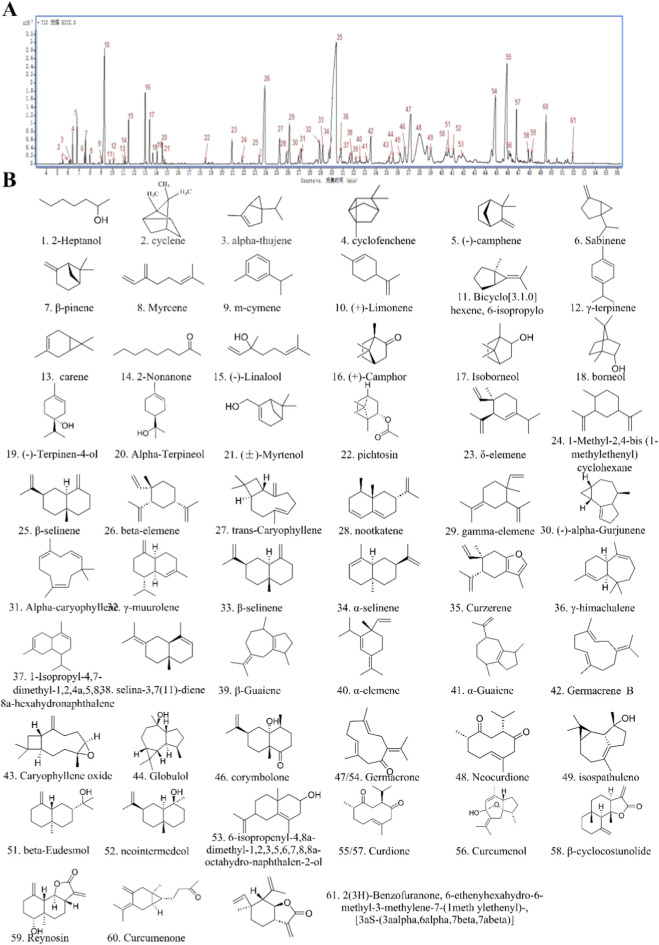
Identification of Bioactive Components in ZTO. **(A)** GC-MS result chromatograms for ZTO. **(B)** Structures of various chemical compounds identified by GC-MS.

Our previous results demonstrated S100A14 as a critical mediator and therapeutic target in BrM, as evidenced by its significant elevation in the peripheral blood of lung cancer patients with BrM and its negative correlation with patient survival. S100A14 overexpression has been proved to promote BrM in both lung and breast cancer models. To investigate whether ZTO inhibited BrM by directly targeting S100A14 and reprogramming astrocytes, we first identified its bioactive chemical constituents using GC-MS analysis. The total ion chromatogram was presented in Figure 6A. Using the TCMSP database and by reviewing the literature, the chemical components of Ezhu were compared with the mass spectrometry data, and a total of 57 compounds were identified in ZTO (Table 2). The most abundant constituents included Curzerene, beta-elemene, (+)-Limonene, Germacrone, and Curdione. The molecular structures of all identified compounds were shown in Figure 6B.

**TABLE 2:** Information of identified compounds in ZTO.

Number	Name	Molecular formula	*m/z*	t_R_ (min)
1	2-Heptanol	C_7_H_16_O	116.201	5.510
2	cyclene	C_10_H_16_	136.234	6.031
3	alpha-thujene	C_10_H_16_	136.234	6.251
4	cyclofenchene	C_10_H_16_	136.234	6.426
5	(−)-camphene	C_10_H_16_	136.234	6.841
6	Sabinene	C_10_H_16_	136.234	7.382
7	β-pinene	C_10_H_16_	136.234	7.662
8	Myrcene	C_10_H_16_	136.234	7.983
9	m-cymene	C_10_H_14_	134.218	9.023
10	(+)-Limonene	C_10_H_16_	136.234	9.341
11	Bicyclo[3.1.0]hexene, 6-isopropylo	C_10_H_16_	136.234	9.854
12	γ-terpinene	C_10_H_16_	136.234	10.073
13	carene	C_10_H_16_	136.234	11.015
14	2-Nonanone	C_9_H_18_O	142.239	11.114
15	(−)-Linalool	C_10_H_18_O	154.250	11.367
16	(+)-Camphor	C_10_H_16_O	152.233	13.046
17	Isoborneol	C_10_H_18_O	154.250	13.394
18	borneol	C_10_H_18_O	154.250	13.735
19	(−)-Terpinen-4-ol	C_10_H_18_O	154.250	14.187
20	Alpha-Terpineol	C_10_H_18_O	154.250	14.648
21	(±)-Myrtenol	C_10_H_16_O	152.233	14.784
22	pichtosin	C_12_H_20_O_2_	196.290	18.462
23	δ-elemene	C_15_H_24_	204.351	20.924
24	1-Methyl-2,4-bis(1-methylethenyl)cyclohexane	C_13_H_22_	178.314	21.835
25	β-selinene	C_15_H_24_	204.351	23.475
26	beta-elemene	C_15_H_24_	204.351	23.893
27	trans-Caryophyllene	C_15_H_24_	204.351	25.254
28	nootkatene	C_15_H_24_	202.170	25.816
29	gamma-elemene	C_15_H_24_	204.351	26.161
30	(−)-alpha-Gurjunene	C_15_H_24_	204.351	27.015
31	Alpha-caryophyllene	C_15_H_24_	204.351	27.019
32	γ-muurolene	C_15_H_24_	204.351	28.793
33	β-selinene	C_15_H_24_	204.351	29.264
34	α-selinene	C_15_H_24_	204.351	29.804
35	Curzerene	C_15_H_20_O	216.319	30.051
36	γ-himachalene	C_15_H_24_	204.351	30.831
37	1-Isopropyl-4,7-dimethyl-1,2,4a,5,8,8a-hexahydronaphthalene	C_15_H_24_	204.351	31.683
38	selina-3,7(11)-diene	C_15_H_24_	204.351	31.912
39	β-Guaiene	C_15_H_24_	204.351	32.239
40	α-elemene	C_15_H_24_	204.351	32.563
41	α-Guaiene	C_15_H_24_	204.351	33.361
42	Germacrene B	C_15_H_24_	204.351	33.596
43	Caryophyllene oxide	C_15_H_24_O	220.350	35.216
44	Globulol	C_15_H_26_O	222.366	35.589
46	corymbolone	C_15_H_24_O_2_	236.350	36.783
47/54	Germacrone	C_15_H_22_O	218.335	37.031
48	Neocurdione	C_15_H_24_O_2_	236.350	38.022
49	isospathuleno	C_15_H_24_O	220.350	39.014
51	beta-Eudesmol	C_15_H_28_O	224.382	40.067
52	neointermedeol	C_15_H_26_O_2_	222.200	41.021
53	6-isopropenyl-4,8a-dimethyl-1,2,3,5,6,7,8,8a-octahydro-naphthalen-2-ol	C_15_H_24_O	220.350	42.012
55/57	Curdione	C_15_H_24_O_2_	236.350	45.841
56	Curcumenol	C_16_H_26_O_2_	250.19	46.126
58	β-cyclocostunolide	C_15_H_20_O_2_	232.318	47.925
59	Reynosin	C_15_H_20_O_3_	248.318	48.427
60	Curcumenone	C_15_H_22_O_2_	234.334	49.582
61	2(3H)-Benzofuranone, 6-ethenyhexahydro-6-methyl-3-methylene-7-(1meth ylethenyl)-,[3aS-(3aalpha,6alpha,7beta,7abeta)]	C_15_H_20_O_2_	232.318	51.956

**FIGURE 7:**
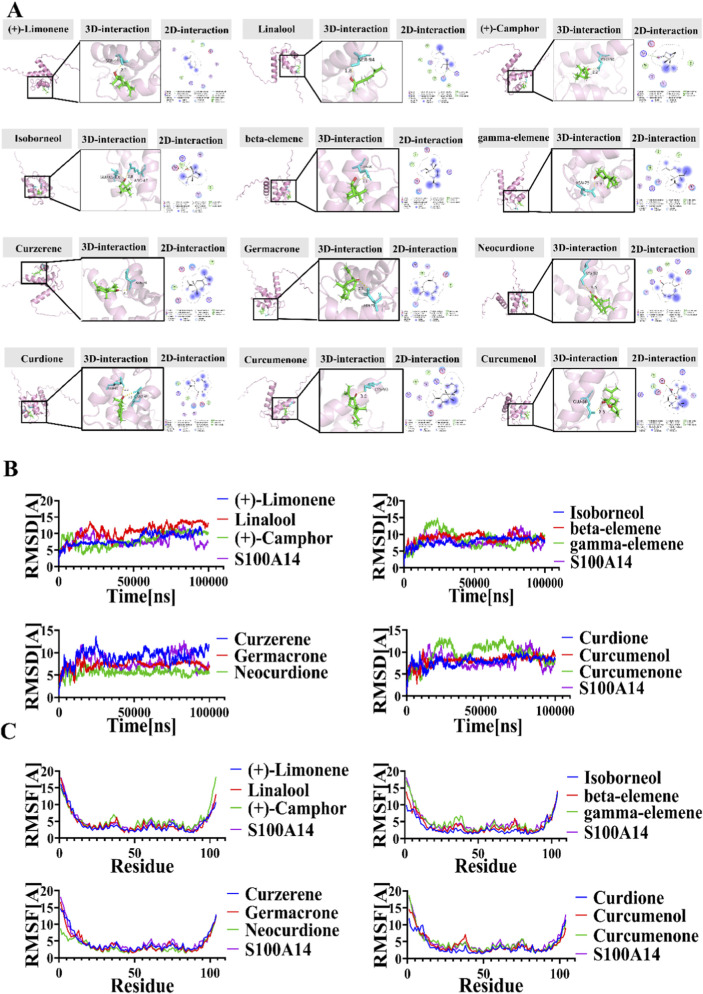
Molecular docking and dynamics simulations predict binding of compounds identified from ZTO and S100A14. **(A)** Molecular docking analysis was used to predict binding affinities between compounds and S100A14. The RMSD **(B)** and RMSF **(C)** results of the MD simulation of compounds in ZTO and S100A14.

Subsequently, molecular docking was employed to evaluate the potential binding affinity of these compounds to S100A14 protein. Molecular docking results indicated that all identified compounds in ZTO exhibited favorable binding energies to S100A14 by extensive hydrogen bonding and hydrophobic interactions (Table 3). Further analysis focused on the top 12 most abundant compounds, including (+)-Limonene, (−)-Linalool, (+)-Camphor, Isoborneol, beta-elemene, gamma-elemene, Curzerene, Germacrone, Neocurdione, Curdione, Curcumenol, and Curcumenone. All these compounds were predicted to bind directly to S100A14, with calculated binding affinities ranging from −4.26 to −5.58 kcal/mol (Figure 7A). The binding interactions involved nine specific residues: SER-44, SER-94, ARG-45, ARG-47, ASN-70, ASN-75, GLU-65, GLU-86, GLU-90, and LYS-93.

**TABLE 3:** Binding energy of S100A14 with components of ZTO.

Number	Compound	Binding energe(Kcal/mol)
1	2-Heptanol	−4.62
2	cyclene	−4.40
3	alpha-thujene	−5.13
4	cyclofenchene	−4.15
5	(−)-camphene	−4.12
6	Sabinene	−4.76
7	β-pinene	−4.32
8	Myrcene	−5.84
9	m-cymene	−4.71
10	(+)-Limonene	−4.96
11	Bicyclo[3.1.0]hexene, 6-isopropylo	−4.33
12	γ-terpinene	−4.63
13	carene	−5.06
14	2-Nonanone	−4.68
15	(−)-Linalool	−4.80
16	(+)-Camphor	−4.35
17	Isoborneol	−4.26
18	borneol	−4.12
19	(−)-Terpinen-4-ol	−4.81
20	Alpha-Terpineol	−4.87
21	(±)-Myrtenol	−4.30
22	pichtosin	−4.36
23	δ-elemene	−5.38
24	1-Methyl-2,4-bis(1-methylethenyl)cyclohexane	−5.17
25	β-selinene	−4.95
26	beta-elemene	−5.17
27	trans-Caryophyllene	−4.84
28	nootkatene	−4.74
29	gamma-elemene	−5.01
30	(−)-alpha-Gurjunene	−4.93
31	Alpha-caryophyllene	−4.58
32	γ-muurolene	−5.02
33	β-selinene	−4.95
34	α-selinene	−5.13
35	Curzerene	−5.55
36	γ-himachalene	−5.31
37	1-Isopropyl-4,7-dimethyl-1,2,4a,5,8,8a-hexahydronaphthalene	−5.17
38	selina-3,7(11)-diene	−5.03
39	β-Guaiene	−4.93
40	α-elemene	−5.00
41	α-Guaiene	−5.27
42	Germacrene B	−4.57
43	Caryophyllene oxide	−4.78
44	Globulol	−4.70
46	corymbolone	−5.59
47/54	Germacrone	−5.20
48	Neocurdione	−4.89
49	isospathulenol	−5.06
51	beta-Eudesmol	−4.39
52	neointermedeol	−5.13
53	6-isopropenyl-4,8a-dimethyl-1,2,3,5,6,7,8,8a-octahydro-naphthalen-2-ol	−5.38
55/57	Curdione	−4.86
56	Curcumenol	−5.58
58	β-cyclocostunolide	−4.77
59	Reynosin	−5.17
60	Curcumenone	−5.09
61	2(3H)-Benzofuranone, 6-ethenyhexahydro-6-methyl-3-methylene-7-(1meth ylethenyl)-,[3aS-(3aalpha,6alpha,7beta,7abeta)]	−5.00

To validate the stability of these predicted complexes, MD simulations were performed. As shown in Figure 7B, the RMSD of the unbound (apo) S100A14 protein (purple curve) remained stable within a range of 6–9 Å throughout the simulation, indicating that the protein itself maintains a stable dynamic equilibrium in the absence of ligand. In contrast, upon compound binding, all complexes displayed distinct RMSD profiles, signifying ligand-induced conformational rearrangements. The RMSD values for the complexes were consistently higher than that of the apo protein. During the initial 20 ns, the RMSD generally increased from around 7–9 Å before converging to stable plateau levels. At the simulation midpoint onward, the RMSD stabilized within a range of 9–15 Å. This pattern suggested that compound binding, mediated by intermolecular non-covalent interactions triggers moderate to significant conformational adjustments in S100A14, ultimately leading to a new dynamic equilibrium. The average RMSD values for the bound complexes ranged from 5.5 ± 1.3 Å nm to 14.8 ± 1.6 Å (n = 3), confirming that the long-term conformational stability post-binding depends on the specific dynamic balance of protein-ligand interactions. Furthermore, RMSF analysis (Figure 7C) revealed that while the N- and C-terminal regions of S100A14 retained high flexibility (RMSF > 15 Å) even after sesquiterpene binding, the secondary structural elements constituting the ligand-binding pocket exhibited markedly increased rigidity (RMSF < 5 Å). This indicated that the ligand selectively stabilizes the functional core of the protein through an anchoring effect, while preserving the dynamic flexibility of the terminal regions, which may be necessary for adapting to downstream interactions or regulatory functions. Collectively, molecular docking, and MD simulation data provide strong *in silico* evidence that multiple bioactive compounds within ZTO can directly and stably bind to S100A14.

**FIGURE 8:**
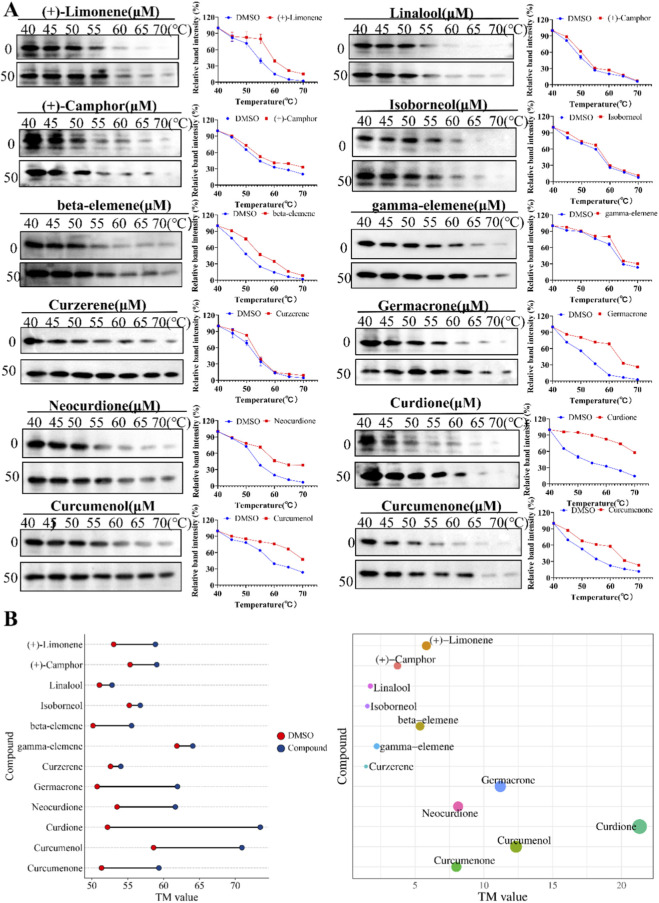
CETSA detect thermal stability of S100A14 in presence of compounds identified from ZTO. **(A)** Representative immunoblots of cell lysates incubated at different temperatures (40, 45, 50, 55, 60, 65 and 70 °C) in the presence of compounds identified from ZTO. **(B)** Graphical representation of the results shown in part A. Data represent mean ± SD (n = 3). Significant difference versus control group, *P < 0.05, **P < 0.01, and ***P < 0.001.

**FIGURE 9:**
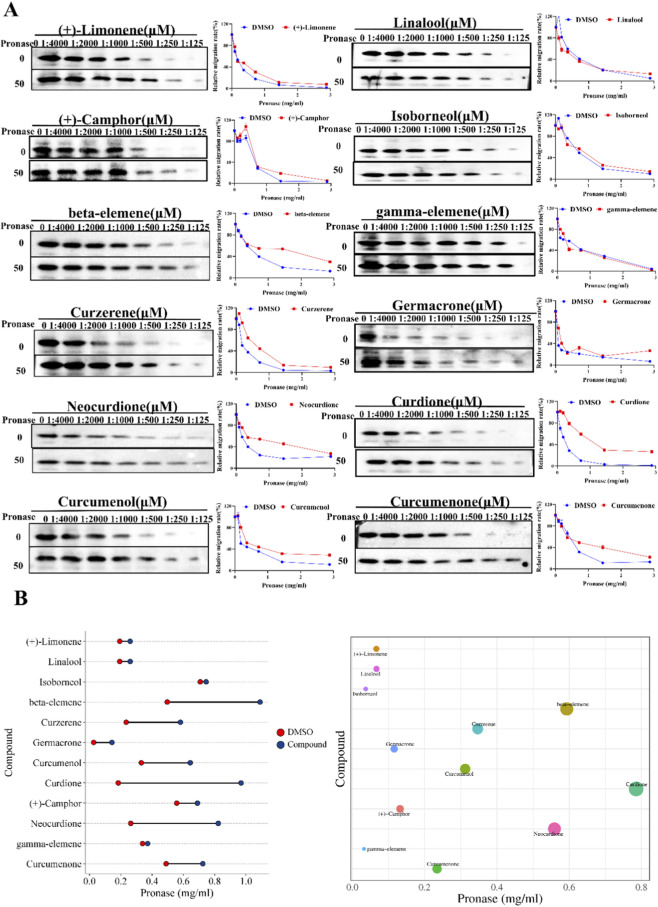
DARTS assay verified the binding between compounds identified from ZTO and S100A14. **(A)** Representative immunoblots of cell lysates incubated at different concentration of pronase (1:4000, 1:2000, 1:1000, 1:500, 1:250, 1:125) in the presence of compounds identified from ZTO. **(B)** Graphical representation of the results shown in part A. Data represent mean ± SD (n = 3). Significant difference versus control group, *P < 0.05, **P < 0.01, and ***P < 0.001.

Finally, CETSA and DARTS assays were performed to validate the interaction. CETSA results confirmed that, as shown in Figure 8A, S100A14 could be established in presence of compounds Curzerene, Germacrone and Curdione, especially at 45 °C, and noticeable results could be observed. Meanwhile, we plotted the relationship S100A14 protein and temperature to generate thermal melting curves, and calculated melting temperatures (Tm, the temperature at which 50% of proteins are unfolded and rapidly precipitated by heat). The average Tm in Curzerene-treated group was increased from 48.5 °C to 70.5 °C. In Germacrone group, Tm value increased from 48.5 °C to 62.7 °C. In Curdione group, Tm value increased from 44.2 °C to 61.9 °C. These results indicated that compounds Curzerene, Germacrone and Curdione triggered thermal stabilization of S100A14 through direct binding. We also found that compounds (+)-Limonene, β-elemene, Neocurdione, Curcumenol and Curcumenone could bound to S100A14, thereby preventing its degradation at 50 °C. Only compounds Linalool, (+)-Camphor, Isoborneol and γ-elemene have no significant effect on the stability of S100A14 protein. Then, we explored DARTS to examined the impact of compounds on the protein stability of S100A14 (Figure 9A). Consistent with the results of CETSA, the results of DARTS also confirmed that the compounds (+)-Limonene, Curzerene, Germacrone, Curdione, Curcumenol and Curcumenone at the concentration of 50 μM could increase the stability of S100A14 during the pronase. Collectively, comprehensive GC-MS analysis identified a total of 57 compounds, including 32 sesquiterpenes, 20 monoterpenes, and five other compounds. Critically, CETSA and DARTS assays confirm that multiple compounds such as (+)-Limonene, Curzerene, Germacrone, Curdione, Curcumenol and Curcumenone, can directly combine with S100A14, thereby increasing the thermal stability and protease hydrolytic stability of S100A14 protein.

## 4 Discussion

BrM remain a challenging clinical problem with a very poor prognosis, highlighting the urgent need to develop new therapeutic strategies. Our study established ZTO, a TCM extract, as a highly promising candidate, demonstrating potent and dose-dependent efficacy against S100A14-driven BrM from both breast and lung cancers mice models. Utilizing intracarotid BrM mice model, we firstly demonstrated that ZTO treatment significantly reduced tumor burden in the brain in a dose-dependent manner, as evidenced by both *in vivo* (Figures 1A,C,H,J) and *in situ* (Figures 1B,D,I,K) BLI. H&E staining analyses consistently revealed a significant suppression of tumor growth and burden in the brains of 200 mg/kg ZTO group, with efficacy superior to the current clinical chemotherapeutic agent, TMZ (Figures 1G,N). Notably, the anti-BrM efficacy of ZTO was achieved without eliciting significant systemic toxicity, as indicated by stable body weight (Figures 1F,M) and organ index (Figures 1E,L) throughout the treatment period. Our results highlighted that ZTO exhibited considerable promise as a novel therapeutic candidate for the treatment of tumor BrM.

Mechanistically, our findings reveal that ZTO inhibited S100A14-driven BrM by reprogramming the brain metastatic microenvironment, primarily through the regulation of GFAP^+^ astrocytes. In the S100A14-induced BrM microenvironment, astrocytes become activated and drive neuroinflammation to create a permissive niche for tumor growth. We found that ZTO treatment markedly attenuated GFAP^+^ astrocyte accumulation around tumor lesions and concurrently suppressed production of key pro-inflammatory cytokines (e.g., IL-6, IL-4) and chemokines (e.g., CCL2, CCL7). The attenuation of pro-inflammatory factors subsequently disrupted the recruitment of P-MDSCs and M-MDSCs into the brain, thereby alleviating the local immunosuppressive niche, which is unfavorable for the survival and outgrowth of tumor cells. Since NF-κB is the main regulator for the inflammatory response in astrocytes, we isolated astrocytes using magnetic beads and examined the NF-κB signaling pathway. We found that ZTO directly inhibited the NF-κB signaling cascade within astrocytes, as evidenced by the reduced phosphorylation of IKKα/β, IκBα, and NF-κB. Therefore, ZTO effectively disrupted an important pro-metastatic signaling axis that promotes tumor BrM, transforming astrocytes from active facilitator of BrM microenvironment formation into quiescent, and reshaping the microenvironment from immunosuppressive to normal.

Our previously research has been confirmed that CM from S100A14-overexpressing tumor cells can significantly enhance the migration and invasion abilities of astrocyte. In this study, we also observed that ZTO treatment directly suppressed S100A14-induced astrocyte migration and invasion in a dose-dependent manner. The migration and invasion inhibitory effect was paralleled by downregulating of key matrix metalloproteinases, MMP-2 and MMP-9. This finding is of great significance because reactive astrocytes play a crucial role in the physical invasion and remodeling of the extracellular matrix, which is essential for the formation and expansion of metastatic lesions. By downregulating MMPs, ZTO not only restrained the invasive capacity of As but may also block the role in facilitating tumor cell dissemination and angiogenesis within the brain. This dual effects of inhibiting NF-κB to suppress the secretion of pro-inflammatory factors and inhibiting MMPs to suppress the migration ability of astrocytes indicated that ZTO can exert its anti-BrM effect on multiple aspects of reactive astrocytes. This two-pronged strategy of ZTO delivery a more comprehensive attack on tumor BrM than therapies targeting a single pathway.

Finally, utilizing an integrated computational and experimental approach, we provided a crucial link between ZTO’s bioactive constituents and S100A14. Molecular docking and dynamics simulations predicted stable, energetically favorable binding of Curdione, Curzerene, Germacrone, and (−)-Linalool to the S100A14 protein, primarily through interactions with key residues like SER-44, GLU-90, ASN-75, LYS-93. Notably, these compounds were predicted to occupy partially overlapping yet distinct binding regions on S100A14: Curzerene and Germacrone preferentially interacted with the hydrophobic pocket formed by SER-44 and ASN-75, while Curdione and (−)-Linalool showed additional contacts with GLU-90 and LYS-93. This suggests that multiple components could simultaneously bind to different domains of the same S100A14 protein, a multi-site binding pattern that may induce cooperative conformational changes and result in more potent functional inhibition than any single compound alone. These *in silico* predictions were robustly validated by our *in vitro* CETSA and DARTS assays, which confirmed that several of these compounds directly bind to S100A14, enhancing increased thermal and proteolytic stability. These results suggested that the multi-components of ZTO is not a drawback but a potential strength, enabling a synergistic and polypharmacological effects on a key driver of the BrM cascade.

Study limitations. While multiple lines of biochemical (CETSA/DARTS), computational (molecular docking and dynamics simulations), and pharmacological evidence support S100A14 as a direct functional target of ZTO, definitive genetic validation via S100A14 knockout and rescue experiments in astrocytes has not been completed in the current study. We acknowledge that such experiments would provide the most conclusive evidence for the on-target specificity of ZTO. These experiments (CRISPR-Cas9-mediated S100A14 knockout in astrocytes, followed by re-expression rescue) are currently underway in our laboratory (plasmid construction and preliminary screening have been initiated) and will be reported separately in our future work. The absence of this genetic validation represents a limitation of the present study. Additionally, two other limitations should be noted: first, the intracarotid injection model, while highly reproducible and suitable for efficacy assessment, does not fully recapitulate the multi-step spontaneous metastasis process; second, although multiple ZTO components were shown to bind S100A14, the specific component(s) responsible for the *in vivo* anti-BrM effect remain to be identified.

## 5 Conclusion

In conclusion, our study delineates a novel anti-BrM mechanism of ZTO: bioactive terpenoids in ZTO directly targets S100A14, thereby inhibiting its function in activating the NF-κB pathway to suppress the release of pro-inflammatory factors in astrocytes, and disrupting the recruitment of MDSCs, ultimately reprogramming the brain’s immune landscape. This study not only provides robust proof that ZTO as a promising anti-BrM therapeutic agent but also establishes a paradigm for developing targeted therapies against the “seed and soil” interaction in BrM.

## Data Availability

The original contributions presented in the study are included in the article/Supplementary Material, further inquiries can be directed to the corresponding authors.
